# Identification and *in silico* Characterization of Deleterious Single Nucleotide Variations in Human ZP2 Gene

**DOI:** 10.3389/fcell.2021.763166

**Published:** 2021-11-17

**Authors:** Neha Rajput, Gagandeep Kaur Gahlay

**Affiliations:** Department of Molecular Biology and Biochemistry, Guru Nanak Dev University, Amritsar, INDIA

**Keywords:** human ZP2, fertilization, SNP, in silico study, female infertility

## Abstract

ZP2, an important component of the zona matrix, surrounds mammalian oocytes and facilitates fertilization. Recently, some studies have documented the association of mutations in genes encoding the zona matrix with the infertile status of human females. Single nucleotide polymorphisms are the most common type of genetic variations observed in a population and as per the dbSNP database, around 5,152 SNPs are reported to exist in the human *ZP2* (*hZP2*) gene. Although a wide range of computational tools are publicly available, yet no computational studies have been done to date to identify and analyze structural and functional effects of deleterious SNPs on *hZP2*. In this study, we conducted a comprehensive *in silico* analysis of all the SNPs found in *hZP2*. Six different computational tools including SIFT and PolyPhen-2 predicted 18 common nsSNPs as deleterious of which 12 were predicted to most likely affect the structure/functional properties. These were either present in the N-term region crucial for sperm-zona interaction or in the zona domain. 31 additional SNPs in both coding and non-coding regions were also identified. Interestingly, some of these SNPs have been found to be present in infertile females in some recent studies.

## 1 Introduction

Reproduction is a fundamental process, which ensures the continued existence of all life forms. Natural selection has preferred sexual reproduction over asexual one, even though it is lengthy and complicated. The reason being its great contribution to genetic diversity, which gives different life forms an upper hand in the race to the survival of the fittest. Sexual reproduction involves the unification of two gametes i.e. sperm and oocyte from the male and female parents respectively during fertilization. Mammalian oocytes are surrounded by an extracellular fibrous matrix called zona pellucida (ZP), which plays an important role in folliculogenesis, fertilization, block to polyspermy, and in the protection of embryo during pre-implantation (Zhao and Dean, 2002). In humans, ZP is composed of four distinct glycoproteins designated as ZP1, ZP2, ZP3, and ZP4 (Lefièvre et al., 2004). Among these constituent proteins, ZP2 plays a significant role in allowing sperm to bind to the unfertilized egg and eventually lead to post-fertilization block to polyspermy (Gahlay et al., 2010; Burkart et al., 2012). Human ZP2 protein (hZP2) is encoded by a single copy gene (*hZP2*) which is located on the 16th chromosome (band: p12.3-p12.2). It consists of 20 exons which encode for 5 mRNA splice variants. Among these, one variant (NM_003460) encodes for the 745 amino acid long protein product (NP_003451) forming the zona matrix. hZP2 is a glycoprotein having both N-linked (∼37% of its molecular weight) as well as O-linked glycosylation (∼8%) (Chiu et al., 2008). The nascent ZP2 protein has an N-terminal signal peptide sequence, a conserved ZP domain, a consensus furin cleavage site, and a C-terminal transmembrane domain (Gupta and Brunak, 2002).

Investigating the relationship between nucleotide variations at the DNA level and the subsequent changes in the structure and function of the associated proteins with the diseased condition is a major challenge for researchers. Single nucleotide polymorphisms (SNPs) are the most common type of genetic variation in humans. Among these, the non-synonymous SNPs (nsSNPs), which result in encoding for a different amino acid, can have drastic effects on protein structure, function, and the associated phenotype. Numerous studies have proven the role of SNPs in different diseased conditions like infectious diseases (Schröder and Schumann, 2005), Type 2 diabetes (Willer et al., 2007) breast cancer (Rajasekaran et al., 2007), polycystic ovary syndrome (PCOS) (Chen and Fang, 2018), male infertility (Zhang et al., 2014; Li et al., 2017), etc. With the availability of Next Generation genome sequencing and different databases like dbSNP, GWAS Central, SwissVar, etc. the presence of SNPs in different genes can be easily studied. Further, to assist genetic studies, several machine learning tools have also recently been developed to identify and predict the impact of variants of unknown significance and pathogenicity (Peterson et al., 2013; Niroula and Vihinen, 2016).

Although SNP analysis for several genes involved in different diseased conditions has been done, yet the role of SNPs in ZP genes in altering its protein’s structure and function and thus, their correlation with female infertility has not been widely studied. Association between SNP’s in ZP genes and fertilization failure in IVF (Männikkö et al., 2005), anomalies in ZP (Pökkylä et al., 2011; Zhou et al., 2019), familial infertility (Huang et al., 2014) have although been recently indicated in some studies. Of the various ZP proteins, ZP2 is critical in the first step of sperm-egg interaction facilitating the binding of sperm with an unfertilized oocyte (Gahlay et al., 2010). Sperm are unable to bind to an oocyte if the N-terminus region (51–149 aa) of ZP2 is absent (Avella et al., 2014). Post-fertilization, cleavage of ZP2 prevents the sperm to bind to the fertilized egg and is thus also involved in the post-fertilization block to polyspermy (Gahlay et al., 2010; Burkart et al., 2012). Considering this, it becomes imperative to study the effect of human *ZP2* (*hZP2*) SNPs on fertility.

For this, the dbSNP database was analyzed and around 5,152 SNPs are reported to exist for our candidate gene h*ZP2* (retrieved as of Dec 2020). No computational study has been done so far to prioritize the deleterious SNPs in the *hZP2* in terms of their disease causing potential. So, this study is aimed to explore the various bioinformatics tools, in order to identify and predict the most deleterious single nucleotide variations in *hZP2* based on their predicted structural, functional, and regulatory effect(s) on the protein. Apart from increasing our existing knowledge in explaining the putative involvement of genetic background in deciding the reproductive fitness of the females, this knowledge can also be used to use these deleterious SNPs in the detection of idiopathic female infertility.

## 2 Materials and Methods

### 2.1 Datasets and SNP Retrieval

**FIGURE 1:**
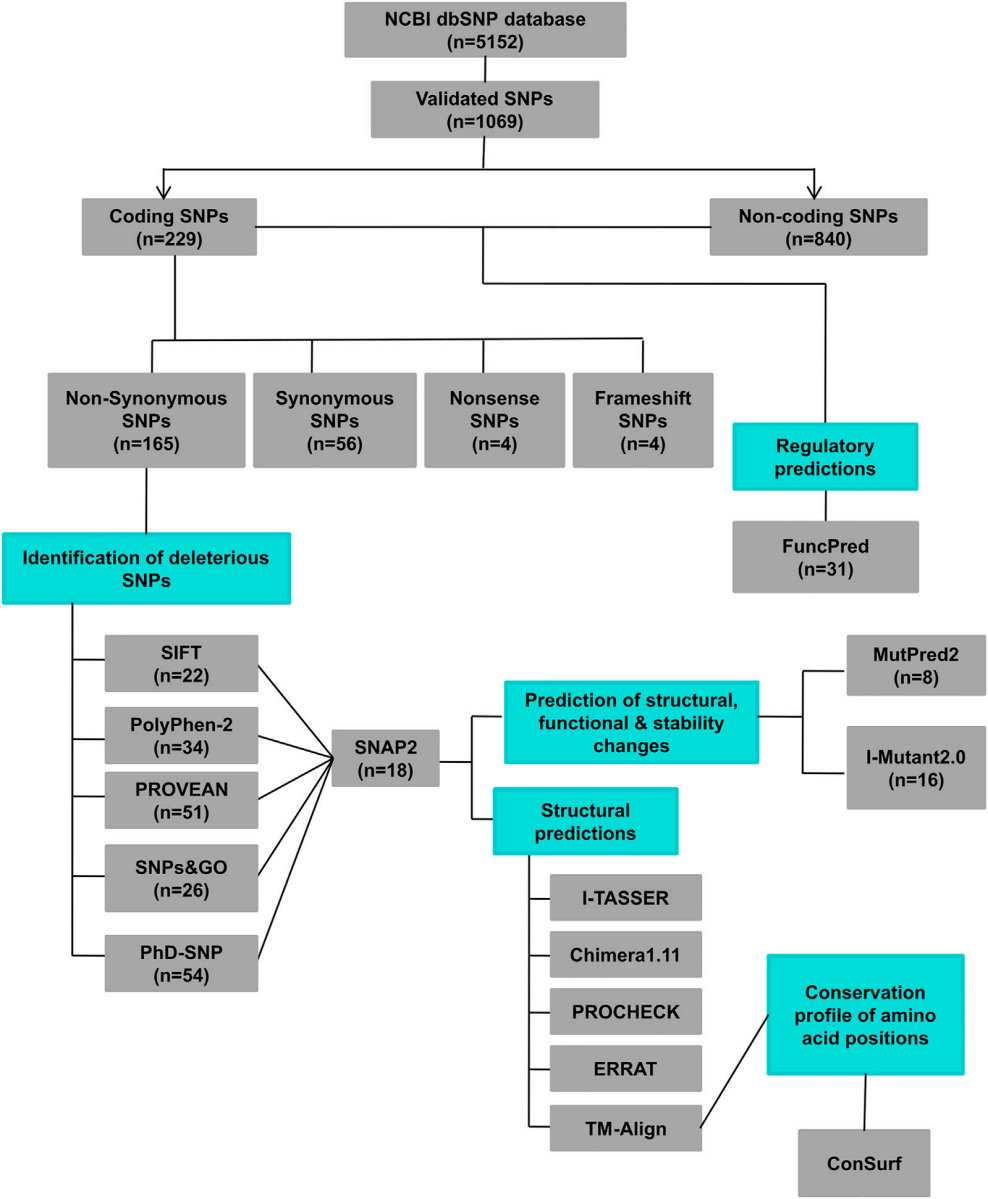
A schematic representation of different *in silico* tools used at different steps of this study (*n* = number of SNPs).

The *hZP2* gene data was obtained from *Entrez* Gene on National Center for Biological Information (NCBI) website and Ensembl genome database. The SNP information for *hZP2* (rsIDs, chromosomal position, residue change, and global minor allele frequency (MAF)) was retrieved from the NCBI dbSNP database. Among all the SNPs present in *hZP2*, the validated ones were cataloged into coding and non-coding. The coding SNPs were further categorized into non-synonymous, synonymous, nonsense, and frameshift. The non-synonymous SNPs were then subjected to a variety of *in silico* tools as shown in Figure 1.

### 2.2 Identification of Deleterious or Disease-Associated nsSNPs

To filter out the deleterious non-synonymous SNPs (nsSNPs), six different bioinformatics tools were employed. These include SIFT (Sorting Intolerant From Tolerant; https://sift.bii.a-star.edu.sg/) (Ng and Henikoff, 2001; Kumar et al., 2009), PolyPhen-2 (Polymorphism phenotyping v2; http://genetics.bwh.harvard.edu/pph2/) (Adzhubei et al., 2010, 2013), PROVEAN (Protein Variation Effect Analyzer; http://provean.jcvi.org/index.php) (Choi et al., 2012; Choi and Chan, 2015), SNPs&GO (http://snps.biofold.org/snps-and-go/snps-and-go.html) (Calabrese et al., 2009; Capriotti et al., 2013), PhD-SNP (Predictor of human Deleterious Single Nucleotide Polymorphisms; http://snps.biofold.org/phd-snp/phd-snp.html) (Capriotti et al., 2006) and SNAP2 (Screening for Non-Acceptable Polymorphism; https://www.rostlab.org/services/snap/) (Hecht et al., 2015). All these algorithms use different approaches to classify a non-synonymous single nucleotide variation as deleterious or not. The inputs for these were given either in the form of rsIDs or amino acid substitutions (AAS) corresponding to all the nsSNPs in *hZP2*. The deleterious nsSNPs which were common in at least 3 or 4 algorithms were chosen for further characterization.

### 2.3 Prediction of Structural and Functional Alterations in hZP2 Protein Caused by Deleterious nsSNPs

MutPred2 (http://mutpred.mutdb.org) was used to predict the structural and functional alterations caused by the deleterious nsSNPs. MutPred2 quantifies the pathogenicity of amino acid substitutions and categorizes them as pathogenic or benign in humans and also predicts their impact on 50 different protein properties (Pejaver et al., 2017). The protein sequence in FASTA format along with the AAS corresponding to the selected nsSNPs was submitted as input in this web server. A *p*-value threshold of 0.05 and a prediction score ranging between 0 and 1 was used. A higher score reflects a higher probability of pathogenicity and the possible alterations in properties were represented as gain/loss of protein structure and/or function.

### 2.4 Predicting the Effect of nsSNPs on the Stability of hZP2 Protein

I-Mutant2.0   (http://folding.biofold.org/i-mutant/i-mutant2.0.html) predicts the change in stability of a protein, upon single point mutation. It is based on the dataset derived from the ProTherm database which is the most inclusive database of thermodynamic experimental data of free energy changes of protein stability upon mutation under different conditions (Capriotti et al., 2005). hZP2 protein sequence in FASTA format and individual AAS corresponding to selected deleterious nsSNPs were given as input and the corresponding change in stability (Reliability Index; RI) and free energy (Kcal/mol; represented as DDG) was obtained. The RI ranges from 0–10 with 10 being the highest in reliability.

### 2.5 3D Modeling of Protein Structure

The 3D model of hZP2 protein was obtained using I-TASSER (https://zhanglab.ccmb.med.umich.edu/I-TASSER/) which is the most advanced protein structure prediction server. It uses LOMETS, a multiple threading approach to identify structural templates, and generates a 3D atomic model by comparing it with structurally similar known proteins (Roy et al., 2010). The amino acid sequence of hZP2 protein in FASTA format was given as input. Out of the top five models generated in output, the one with the highest C-score was selected. The model thus selected was viewed in Chimera 1.11 (Pettersen et al., 2004) which allows interactive visualization and analysis of molecular structures and related data. For looking at the effect of SNPs, all the deleterious amino acid changes were substituted manually using its rotamer function, and any new network of contacts or clashes formed were assessed. In addition, 12 mutant models of hZP2 were also generated using I-TASSER by manually substituting the amino acids in FASTA sequence of hZP2 at positions corresponding to the 12 deleterious SNPs.

### 2.6 Quality Assessment of the 3D Model Generation

PROCHECK (https://servicesn.mbi.ucla.edu/PROCHECK/) was used to assess the quality of the 3D model that was generated above. This program gives an evaluation of the overall quality of the structure, based on various stereochemical properties (like phi-psi angles in most favored regions of Ramachandran plot, side-chain parameters, chi1-chi2 plots, etc.) by comparing them with the well-refined protein structures of the same resolution and also highlights the regions that may need further investigation (Laskowski et al., 1993). The selected model of hZP2 in PDB format was submitted as input. The 3D model was also verified by ERRAT (https://servicesn.mbi.ucla.edu/ERRAT/) (Colovos and Yeates, 1993) which compares the statistics of non-bonded interactions of different atoms of the submitted protein model with those of highly refined structures.

TM-Align (https://zhanglab.ccmb.med.umich.edu/TM-align/) was used to calculate TM-score and RMSD values of wild type and mutant models. TM-score tells about the topological similarity between wild type and mutant models and RMSD helps in measuring the average distance between alpha-carbon backbones of wild type and mutant models (Zhang and Skolnick, 2005). The TM-score varies between 0 and 1, with a value of one representing a perfect match between two structures. The RMSD value, on the other hand, represents the deviation of mutant structure from the wild type. A higher RMSD value means greater deviation.

### 2.7 Predicting the Conservation Score of Amino Acid Positions Corresponding to Deleterious nsSNPs

Since the evolutionarily conserved positions in a protein are considered important in terms of its structure and function, the conservation score of all the amino acid positions corresponding to deleterious nsSNPs was calculated using ConSurf (https://consurf.tau.ac.il/) (Ashkenazy et al., 2010). This bioinformatics tool uses PSI-BLAST, CSI-BLAST, or BLAST to find the homologous sequences for the given input sequence and performs multiple sequence alignment using different programs like MAFFT, PRANK, TCOFFEE, MUSCLE, or CLUSTALW and finally gives output in the form of a score that ranges from one to nine where nine represents most conserved and one represents highly variable amino acid position.

### 2.8 Predicting the Putative N and O Glycosylation Sites in hZP2

Since the glycosylation sites on native human zona are not known, we used prediction software to determine this. Potential N- and O-glycosylation sites in the full-length hZP2 (1–745 aa) were predicted using NetNGlyc-1.0 (http://www.cbs.dtu.dk/services/NetNGlyc/) and NetOGlyc-4.0 (http://www.cbs.dtu.dk/services/NetOGlyc/) respectively. NetNGlyc-1.0 predicts N-linked glycosylation sites in human proteins using artificial neural networks that examine the sequence context of Asn-Xaa-Ser/Thr sequons (Gupta R, 2002). NetOGlyc-4.0 predicts O-GalNAc (mucin type) glycosylation sites in mammalian proteins using neural network predictions (Steentoft et al., 2013). Boja et al., 2003 have characterized the N- and O-linked glycosylation sites in mouse ZP2 (mZP2) using mass spectrometry of native mouse zona proteins. Using this data, manual assertions were made for glycosylation in hZP2 by aligning hZP2 and mZP2 protein sequences using Clustal Omega (Madeira et al., 2019). These manual assertions were compared with those predicted by NetNGlyc-1.0 and NetOGlyc-4.0. In addition, to analyze any deviation in terms of loss or gain of N- or O-glycosylation sites caused by the shortlisted 12 deleterious nsSNPs, FASTA sequences corresponding to the polymorphisms were analyzed using NetNGlyc-1.0 and NetOGlyc 4.0 respectively. This was done to ascertain if a change in N- or O-glycosylations resulted in altered interaction with sperm or**,** modified the zona structure.

### 2.9 Functional Predictions of Both Coding and Non-Coding SNPs

To predict the functional effect of both coding and non-coding SNPs, FuncPred (https://snpinfo.niehs.nih.gov/snpinfo/snpfunc.html) was used. This web-based server selects the SNPs from Genome Wide Association Studies (GWAS) and uses GWAS-SNP *p*-value data to predict the effect of SNPs on functional characteristics like splice sites, Transcription factor binding sites (TFBS), microRNA binding sites, etc. (Xu and Taylor, 2009). The rsIDs of all the validated nsSNPs (coding and non-coding) in the *hZP2* gene were used as input and predictions were obtained.

## 3 Results

### 3.1 Retrieval of SNP Dataset From dbSNP Database

According to the dbSNP database, a total of 5,152 SNPs were reported in the human *ZP2* gene (transcript ID: NM_003460 and protein ID: NP_003451). Out of these 5,152, only 1,069 were found to be validated. Further, among the validated, 229 SNPs were in the coding region and the remaining 840 were in the non-coding region (3’ & 5′ near gene region, 3’ & 5’ UTRs, and introns). Among the 229 coding SNPs, 165 were non-synonymous SNPs (missense; nsSNPs), 56 were synonymous (same-sense; sSNPs), four were nonsense, and four were frameshift (insertions and deletions).

### 3.2 18 nsSNPs in the *hZP2* Gene Were Predicted to Be Deleterious

nsSNPs produce amino acid allelic variants of the gene which may affect the structure and function of the protein. Hence, the 165 nsSNPs were selected for further investigations. Of those 22 were predicted to be deleterious by the SIFT web server, with a SIFT score of ≤0.05 (Supplementary Table S1 **)**. Two of the nsSNPs, rs374388107 and rs267604453, had a score of 0 which is considered the most damaging score. Another program, PolyPhen-2 predicted 64 nsSNPs as probably/possibly damaging. Out of these 64, 30 were marked as “probably damaging” (PolyPhen score between 0.950 and 1). The remaining 34 nsSNPs were designated as “possibly damaging” (PolyPhen score between 0.850 and 0.950) (Supplementary Table S2). PROVEAN web server predicted a total of 51 out of 165 nsSNPs as deleterious, with a PROVEAN score of less than the cut-off value (-2.5) (Supplementary Table S3). According to the predictions by another algorithm SNPs&GO, only 26 nsSNPs are found to be disease-associated as they had a probability value of >0.50 which predicts disease association (Supplementary Table S4). PhD-SNP prediction classified 54 nsSNPs as disease-associated (Supplementary Table S5).

To effectively select the most deleterious nsSNPs and reduce the rate of false-positive predictions, we shortlisted 18 nsSNPs which were commonly classified as deleterious in at least 3 or 4 out of the above mentioned five algorithmic tools by manual concordance (Table 1). These were classified as deleterious nsSNPs. These deleterious nsSNPs were cross-validated for having an effect or being a neutral variant using another web-server called SNAP2, which predicted the functional effects caused by these nsSNPs. All the 18 nsSNPs were predicted as “effect variants” with highly expected accuracy, making these good candidates for further investigation (Supplementary Table S6).

**TABLE 1:** List of 18 deleterious nsSNPs in hZP2 gene, as identified by five different *in silico* tools. The score or probability for each is mentioned within brackets.

S.No	rsIDs	Residue change	SIFT prediction (score)	PolyPhen2 (score)	PROVEAN (cutoff = -2.5) (Score)	SNPs&GO (probability)	PhD-SNP (probability)
1	rs199927753	P47H	Deleterious (0.006)	Probably damaging (0.998)	Deleterious (−3.168)	Neutral (0.377)	Disease (0.780)
2	rs559249999	P50S	Not found	Possibly damaging (0.894)	Deleterious (−4.240)	Disease (0.679)	Disease (0.748)
3	rs761335280	C155Y	Not found	Probably damaging (1)	Deleterious (−8.190)	Disease (0.754)	Disease (0.845)
4	rs369091148	G282E	Deleterious (0.003)	Probably damaging (1)	Deleterious (−5.977)	Disease (0.695)	Disease (0.722)
5	rs200645879	Q374H	Deleterious (0.045)	Probably damaging (1)	Deleterious (−3.468)	Neutral (0.288)	Neutral (0.388)
6	rs774816416	G376A	Not found	Probably damaging (1)	Deleterious (−5.569)	Disease (0.576)	Disease (0.666)
7	rs778652791	V382F	Not found	Probably damaging (1)	Deleterious (−4.574)	Disease (0.599)	Disease (0.748)
8	rs144403520	S384I	Deleterious (0.005)	Probably damaging (0.957)	Deleterious (−3.800)	Disease (0.598)	Disease (0.500)
9	rs765444754	P420T	Not found	Probably damaging (1)	Deleterious (−6.797)	Disease (0.621)	Disease (0.588)
10	rs768663589	G425R	Not found	Probably damaging (1)	Deleterious (−7.425)	Disease (0.842)	Disease (0.926)
11	rs141585544	N439K	Tolerated (1)	Probably damaging (1)	Deleterious (−5.491)	Disease (0.805)	Disease (0.903)
12	rs267604453	E440K	Deleterious (0)	Probably damaging (1)	Deleterious (−3.644)	Disease (0.688)	Disease (0.534)
13	rs374388107	T462I	Deleterious (0)	Probably damaging (0.999)	Deleterious (−4.579)	Neutral (0.361)	Neutral (0.294)
14	rs199896192	L531Q	Deleterious (0.001)	Possibly damaging (0.907)	Deleterious (−4.879)	Disease (0.667)	Disease (0.728)
15	rs764770086	A547V	Not found	Probably damaging (1)	Deleterious (−3.661)	Disease (0.623)	Disease (0.734)
16	rs145769990	P553L	Deleterious (0.046)	Probably damaging (0.985)	Deleterious (−8.892)	Disease (0.719)	Disease (0.705)
17	rs140925075	G581S	Deleterious (0.015)	Probably damaging (0.937)	Deleterious (−2.715)	Neutral (0.307)	Neutral (0.289)
18	rs376154774	S627Y	Deleterious (0.03)	Probably damaging (0.977)	Deleterious (−3.344)	Neutral (0.072)	Disease (0.571)

SIFT score: Deleterious ≤0.05 and Tolerated >0.05; PolyPhen-2 score: probably damaging = 0.950–1, possibly damaging = 0.850–0.950, benign = 0; PROVEAN score: deleterious ≤ −2.5; neutral > −2.5; SNPs and GO probability value: disease ≥0.50, neutral <0.50; PhD-SNP probability value: disease ≥0.50, neutral <0.50.

### 3.3 Prediction of Functional and Structural Modifications of Deleterious nsSNPs on hZP2 Protein

Of the 18 deleterious nsSNPs submitted to MutPred2 web-server, only eight were found to score more than 0.50 and thus, were predicted to result in structural and functional alterations like change in stability of protein, gain or loss of relative solvent accessibility, loss of disulfide linkage, loss of DNA binding sites, loss of strand, an altered transmembrane protein, altered metal-binding site, etc. (Table 2). Apart from MutPred2, I-Mutant predicted the effect of these mutations on the stability of hZP2 protein. A decrease in stability was observed for 16 out of the 18 nsSNPs. The resultant free energy change (kcal/mol) and the reliability index for each of the substitutions are shown in Table 3.

**TABLE 2:** List of eight deleterious nsSNPs and the resulting structural and functional alterations in hZP2 protein, as predicted by MutPred2.

S.No	rsIDs	Substitution	MutPred2 score	Alterations
1	rs761335280	C155Y	0.755	Altered transmembrane protein; altered disordered interface; altered stability
2	rs369091148	G282E	0.693	Altered ordered interface; gain of relative solvent accessibility; gain of loop; loss of strand; altered transmembrane protein; altered metal binding
3	rs765444754	P420T	0.687	Altered metal binding; altered transmembrane protein and gain of disulfide linkage at C424
4	rs768663589	G425R	0.649	Altered ordered interface; altered metal binding; altered transmembrane protein; gain of ADP-ribosylation at G425 and disulfide linkage at C424
5	rs141585544	N439K	0.895	Altered transmembrane protein; altered ordered interface altered metal binding; loss of strand; altered DNA binding; altered stability; loss of catalytic site at N439
6	rs374388107	T462I	0.517	Altered transmembrane protein; altered ordered interface; altered metal binding; loss of disulfide linkage at C465
7	rs199896192	L531Q	0.550	Altered transmembrane protein; gain of ADP-ribosylation at R533; altered stability
8	rs764770086	A547V	0.715	Altered transmembrane protein; altered metal binding site; altered ordered interface; loss of disulfide linkage at C545; loss of relative solvent accessibility

**TABLE 3:** I-Mutant2.0 results for the selected 18 deleterious nsSNPs.

S.No	rsIDs	Substitution	Stability	Reliability index	Free energy change (Kcal/mol)
1	rs199927753	P47H	Decrease	9	−2.25
2	rs559249999	P50S	Decrease	9	−2.10
3	rs761335280	C155Y	Decrease	0	−0.23
4	rs369091148	G282E	Increase	4	−0.17
5	rs200645879	Q374H	Decrease	6	−1.04
6	rs774816416	G376A	Decrease	5	−0.47
7	rs778652791	V382F	Decrease	8	−1.77
8	rs144403520	S384I	Increase	5	−0.33
9	rs765444754	P420T	Decrease	9	−1.85
10	rs768663589	G425R	Decrease	9	−1.78
11	rs141585544	N439K	Decrease	3	−0.03
12	rs267604453	E440K	Decrease	7	−0.82
13	rs374388107	T462I	Decrease	5	−1.43
14	rs199896192	L531Q	Decrease	9	−2.68
15	rs764770086	A547V	Decrease	1	−1.05
16	rs145769990	P553L	Decrease	2	−0.19
17	rs140925075	G581S	Decrease	8	−1.31
18	rs376154774	S627Y	Decrease	3	−1.11

### 3.4 Analysis of the Effect of Deleterious nsSNPs on the Structure and Function of hZP2 Protein

**FIGURE 2:**
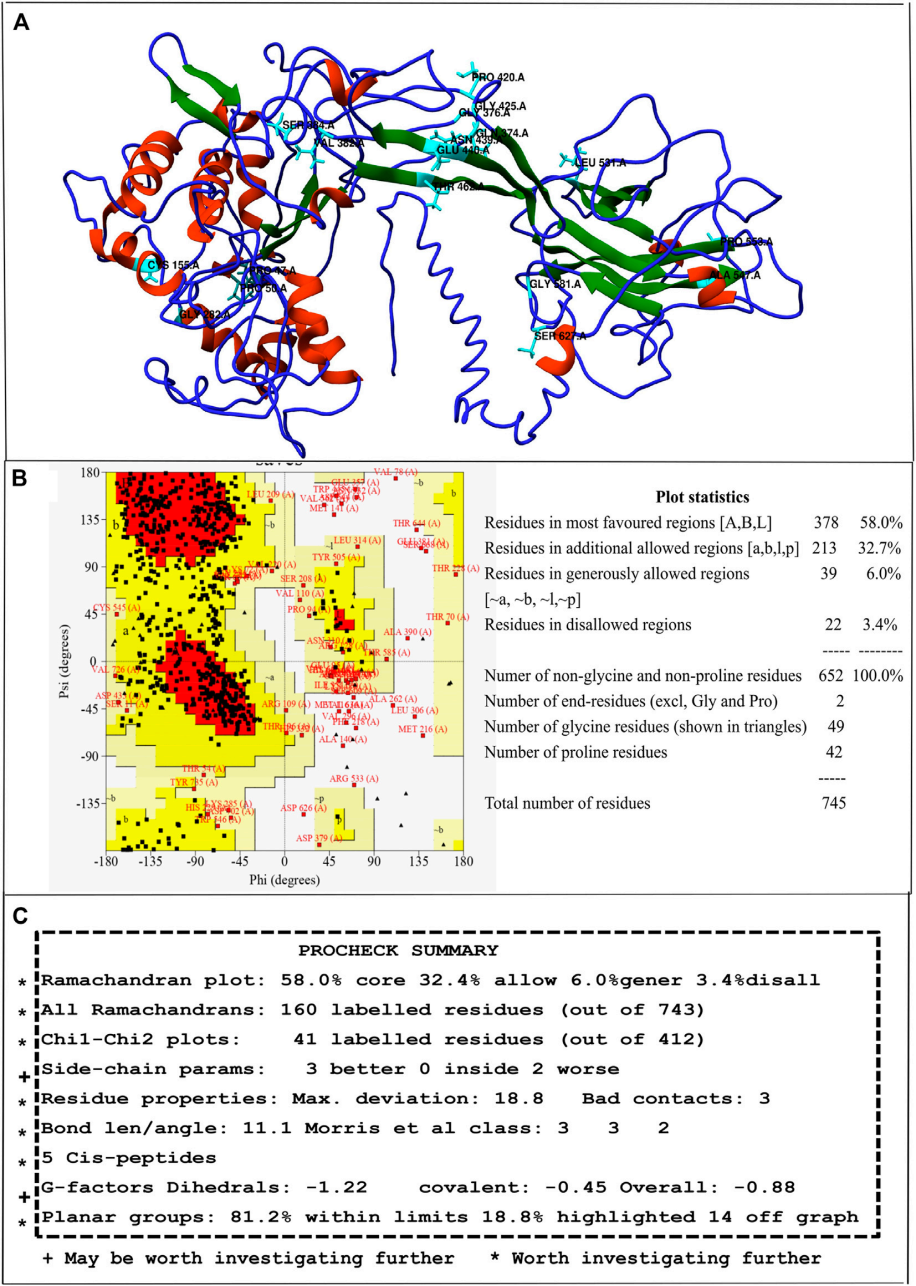
Full-length 3D model of hZP2 generated by I-TASSER. **(A)** Helix regions are shown in red, strand in blue, and coil in green. The positions of 18 deleterious nsSNPs are highlighted in cyan. **(B)** Ramachandran plot for the 3D model of hZP2 **(C)** Screenshot showing the summary of PROCHECK results.

For predicting structural alterations caused due to nsSNPs, first, the 3D model of wild type hZP2 was generated using I-TASSER. Out of the five models generated, the model with the highest C-score of -1.76, an estimated TM-score of 0.50 ± 0.15, and an estimated RMSD of 12.5 ± 4.3Å was used for further analysis **(** Figure 2A **)**. The stereo-chemical quality of the protein model was checked using the PROCHECK program based on various factors like overall G-factor, phi-psi angles, chi1-chi2 plots, side-chain parameters, etc. which were found to be within limits and thus the structure was found acceptable and worth investigating further (Figure 2B). Ramachandran plot showed 58.0% residues in the core region, 32.7% in the allowed region, 6.0% in the generously allowed region, and 3.4% in the disallowed region **(** Figure 2C **)**. ERRAT2 program which verifies the quality of the protein model based on non-bonded interactions predicted the overall quality factor to be 70.21. The generally accepted range for a high-quality model is > 50.

**FIGURE 3:**
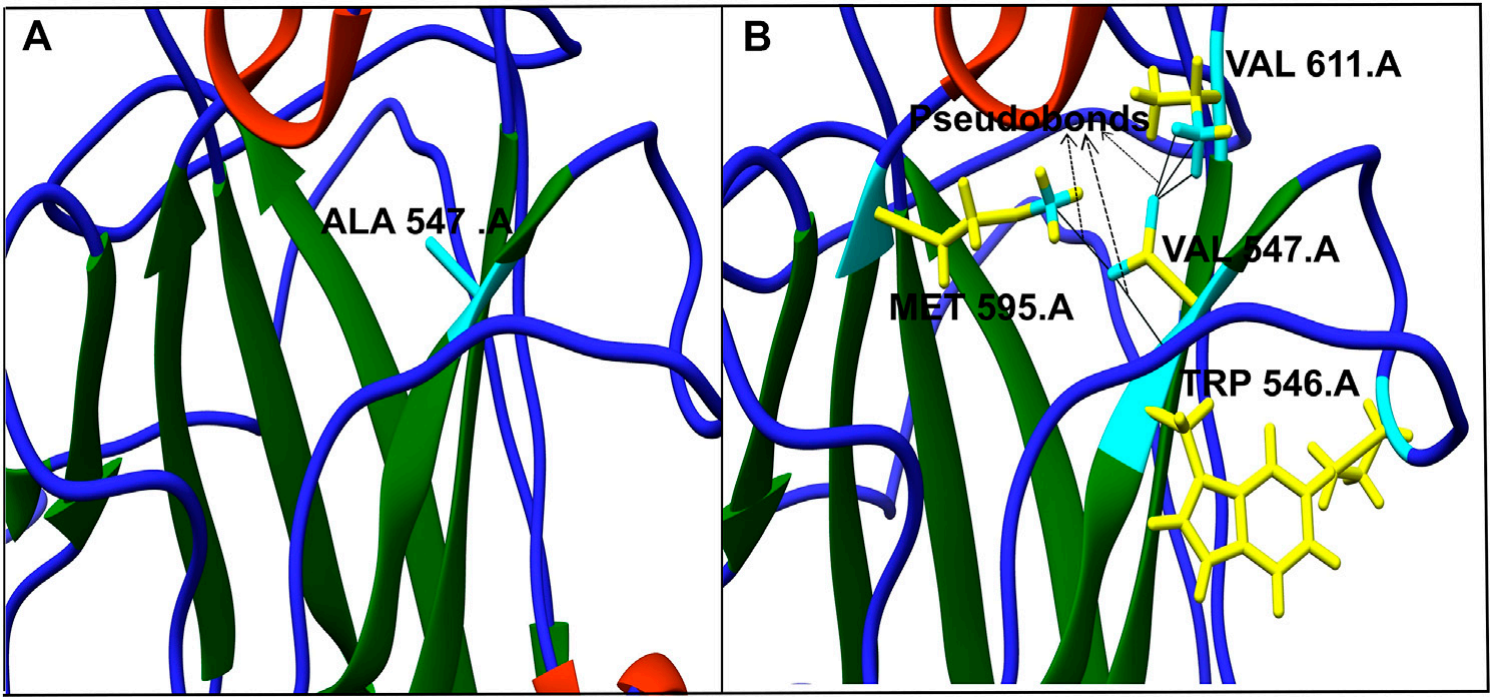
Effect of single nucleotide variation rs764770086 (A547V) on protein’s 3D structure. Alanine at position 547 (cyan) in wild protein forms no contacts in native form **(A)** whereas Valine at the same position (yellow and cyan) forms five new contacts with Val611, Met595, and Trp546 **(B)**. The networks of clashes or new pseudobonds are shown as black lines.

The model generated above was used to study the effect of the mutations on the protein’s 3D structure using Chimera 1.11. Each of the 18 nsSNPs shortlisted above was checked to identify the formation of any new network of contacts and/clashes (Table 4). Out of the 18 nsSNPs, 12 were found to form new networks of clashes and contacts and these were used for further analysis. An example of this is shown in Figure 3 in which, Alanine^547^ formed no network of clashes/contacts in wild type hZP2. However, when it was replaced by Valine, five new pseudo bonds with Val^611^, Met^595,^ and Trp^546^ were formed.

**TABLE 4:** List of 18 deleterious SNPs along with the possible new network of clashes/contacts formed by the substituted amino acids at the corresponding positions as predicted by Chimera 1.11.

S.No	rsIDs	Substitution	Affect	New network of contacts/clashes
1	rs199927753	P47H	√	His at 47 forms 5 new contacts with Leu^44^
2	rs559249999	P50S	X	Serine at 50 forms no new contact
3	rs761335280	C155Y	√	Tyr at position 155 forms 4 new contacts with Gly^112^& Ala^151^
4	rs369091148	G282E	X	Glu at position 282 forms no new contact
5	rs200645879	Q374H	√	His at position 374 forms 1 contact with Asp^375^
6	rs774816416	G376A	X	Ala at position 376 forms no new contact
7	rs778652791	V382F	√	Phe at position 382 forms 10 new contacts with Lys^340^
8	rs144403520	S384I	√	Ile at 384 forms 12 new contacts with Ile^354^, Lys^340^ and Leu^341^
9	rs765444754	P420T	X	Tyr at position 420 forms no new contact
10	rs768663589	G425R	√	Arg at 425 forms 16 new contacts with Glu^399^ and Asn^400^
11	rs141585544	N439K	√	Lys at position 439 forms 8 new contacts with Ile^419^ and Phe^377^
12	rs267604453	E440K	√	Lys at position 440 forms 1 new contact with Arg^460^
13	rs374388107	T462I	X	Ile at position 462 forms no new contact
14	rs199896192	L531Q	√	Gln at position 531 forms 3 new contacts with Thr^494^
15	rs764770086	A547V	√	Val at position 547 forms 5 new contacts with Val^611^, Met^595^ and Trp^546^
16	rs145769990	P553L	√	Leu at position 553 forms 14 new contacts with His^614^ and Leu^656^
17	rs140925075	G581S	X	Ser at position 581 forms no new contact
18	rs376154774	S627Y	√	Tyr at position 627 forms 1 new contact with Asp^626^

√ = Forming new contacts/clashes, X = Not forming any new contact/clash.

### 3.5 Comparative Modeling of Wild Type and Mutant hZP2 Protein

Structural models for the 12 nsSNPs which formed new network of clashes and contacts were also generated using I-TASSER. Out of five models obtained in output for each of the 12 mutant proteins, models with the highest C-score were selected for further analysis. Finally, the wild-type and mutant models were compared using TM-align. The TM-Score and RMSD values for each mutant model are shown in Table 5. Mutant models for C155Y (rs761335280) and S384I (rs768663589) were found to have the lowest TM-score i.e. 0.22707 and 0.20968 and a high RMSD value i.e. 8.27 and 8.44 respectively, thus showing a greater deviation from wild type protein. When the conservation score of 12 amino acid positions (i.e. corresponding to 12 deleterious nsSNPs) on the protein was calculated using ConSurf, it was found that six out of 12 nsSNPs (C155Y, G425R, N439K, E440K, A547V, and S627Y) are in highly conserved positions, thus, showing their functional significance (Table 5 **)**.

**TABLE 5:** TM-align and ConSurf predictions.

S.No	rsID	Substitution	TM-score	RMSD	ConSurf (Score)
1	rs199927753	P47H	0.78370	4.49	Exposed (1)
2	rs761335280*	C155Y	0.22707	8.27	Buried and Highly conserved (9)
3	rs200645879	Q374H	0.80480	4.09	Exposed (6)
4	rs778652791	V382F	0.7844	4.41	Buried (7)
5	rs144403520	S384I	0.87603	3.22	Exposed (7)
6	rs768663589*	G425R	0.20968	8.44	Exposed and Highly conserved (9)
7	rs141585544	N439K	0.70496	3.68	Exposed and Highly conserved (9)
8	rs267604453	E440K	0.77840	3.89	Buried and Highly conserved (9)
9	rs199896192	L531Q	0.75460	4.27	Buried (8)
10	rs764770086	A547V	0.86956	3.48	Buried and Highly conserved (9)
11	rs145769990	P553L	0.86384	3.67	Exposed (6)
12	rs376154774	S627Y	0.79299	3.62	Exposed and Highly conserved (8)

*SNPs having lowest TM-score, highest RMSD value, and are in highly conserved positions.

### 3.6 Effect of SNPs on the Glycosylation Status of hZP2

**FIGURE 4:**
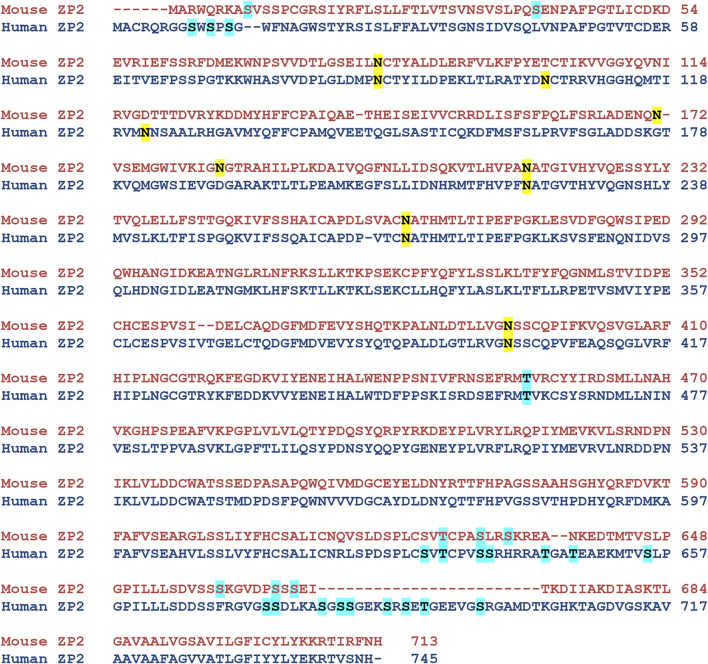
Prediction of N- and O-linked glycosylation sites in hZP2 protein. NetNGlyc-1.0 and NetOGlyc-4.0 were used to predict N-linked and O-linked glycosylation sites in hZP2 respectively. An additional O-glycosylation site at T^462^, based on the data from native mouse ZP2 mass spectrophotometric studies, was also assigned manually. The N-linked and O-linked glycosylation sites in mouse and human ZP2 are highlighted in yellow and cyan respectively.

NetNGlyc predicted 6 amino acid positions in the wild-type hZP2 protein (N^87^, N^105^, N^122^, N^223^, N^269^, and N^400^). When these were compared with the N glycosylation sites characterized in native mZP2, four of these (N^87^, N^223^, N^269^, and N^400^) were present in mouse too. The other two positions (N^105^ and N^122^) were specific to hZP2 (Figure 4). Out of the 12 deleterious nsSNPs, none of them was present at a predicted N-glycosylation site or caused any change in the N-glycosylation pattern due to this polymorphism.

NetOGlyc predicted 19 potential O-glycosylation sites (S^9^, S^11^, S^13^, S^631^, T^633^, S^637^, S^638^, T^644^, T^647^, S^655^, S^674^, S^675^, S^680^, S^682^, S^683^, S^687^, S^689^, T^691^, and S^697^). In addition, based on manual assertion after comparing with mouse mass spectrophotometric data, Thr^462^ was also assigned to be potentially glycosylated in hZP2 (Figure 4). O-linked glycosylation occurs by transferring oligosaccharides to serine and threonine residues. Among the 12 nsSNPs, only two SNPs (S384I and S627Y) translated into the amino acid substitutions where serine was being replaced. However, none including the above two were present at the predicted O-glycosylation sites. When the corresponding mutant sequence for these SNPs was analyzed by NetOGlyc-4.0, loss of glycosylation sites was observed only for C155Y (S^655^), V382F (S^674^, S^691^), N439K (S^655^), E440K (S^691^), L531Q (S^655^), A547V (S^674^, S^682^), P553L (S^655^, S^674^) and S627Y (S^631^, S^674^, S^691^). No loss/gain of O-glycosylation was observed for P47H, Q374H, S384I, and G425R.

### 3.7 31 SNPs Are Predicted to Affect *hZP2* Gene Regulation

The 1,069 validated SNPs from both coding and non-coding region were also submitted to FuncPred to detect their role in the regulation of gene expression. Only 31 of these were found to have an effect. Five coding SNPs (rs16971234, rs2075520, rs2075526, rs34159042, and rs35162028) were found to affect splicing [exonic splicing enhancers (ESE) and exonic splicing silencers (ESS)] and the remaining 26 SNPs from the non-coding region were found to affect transcription factor binding sites (TFBS). Also, no SNP in the 3′UTR region was found to create or abolish the miRNA binding site. The detailed results are shown in Table 6. It is interesting to find that most of these regulatory SNPs have high global MAF values (Supplementary Figure S1).

**TABLE 6:** FuncPred results showing 31 SNPs predicted to affect the *hZP2* gene regulation.

S.No	rsIDs	Location on gene	Global MAF	Effect
1	rs11859854	Intron	ND	TFBS
2	rs11861135	5’ UTR	0.2099	TFBS
3	rs11863124	5’near gene region	0.0006	TFBS
4	rs12598328	5`near gene region	0.0048	TFBS
5	rs13337812	Intron	0.3003	TFBS
6	rs16971234	Exon 14	0.2831	Splicing (ESE & ESS)
7	rs2066755	Intron	-	TFBS
8	rs2075519	Intron	0.2967	TFBS
9	rs2075520	Exon 18	0.4481	Splicing (ESE & ESS)
10	rs2075521	5`UTR	0.4429	TFBS
11	rs2075526	Exon 12	0.3181	Splicing (ESE & ESS)
12	rs28472578	Intron	0.1198	TFBS
13	rs28686859	Intron	0.0262	TFBS
14	rs34159042	Exon 1	0.0002	Splicing (ESE &ESS)
15	rs35162028	Exon 14	0.014	Splicing (ESE & ESS)
16	rs3759984	Intron	0.2973	TFBS
17	rs3759985	Intron	0.3185	TFBS
18	rs3759986	Intron	0.2552	TFBS
19	rs3826157	Intron	0.3045	TFBS
20	rs59018614	5’ near gene region	0.0084	TFBS
21	rs6497541	5`near gene region	0.1699	TFBS
22	rs7187567	Intron	0.1278	TFBS
23	rs7199890	5`near gene	ND	TFBS
24	rs8044116	Intron	0.3844	TFBS
25	rs8053098	Intron	0.0028	TFBS
26	rs8056986	5near gene region	0.0028	TFBS
27	rs8057529	5`near gene region	0.1675	TFBS
28	rs8058730	Intron	0.0128	TFBS
29	rs8064027	Intron	0.4139	TFBS
30	rs9921849	5’near gene region	0.2324	TFBS
31	rs9928409	5`near gene region	0.2418	TFBS

TFBS, Transcription factor binding site.

## 4 Discussion

ZP2 is an important constituent of the zona matrix as *ZP2* null female mice produce zona deficient oocytes and are infertile (Rankin et al., 2001). Using transgenic studies, it has been shown that the cleavage status of ZP2 determines if the egg will be recognized by sperm or not and thereafter prevent polyspermy (Gahlay et al., 2010). Any change in the amino acid sequence of ZP2 protein may affect its structural and functional properties and can thereby affect the ability of the egg to fuse with sperm and/or prevent polyspermy. This can impact the reproductive fitness of mammalian females. These changes in the amino acid sequence of ZP2 may exist naturally in any population in the form of SNPs at the genomic level and result in either a gain of function, loss of function, or no change to the protein.

**FIGURE 5:**
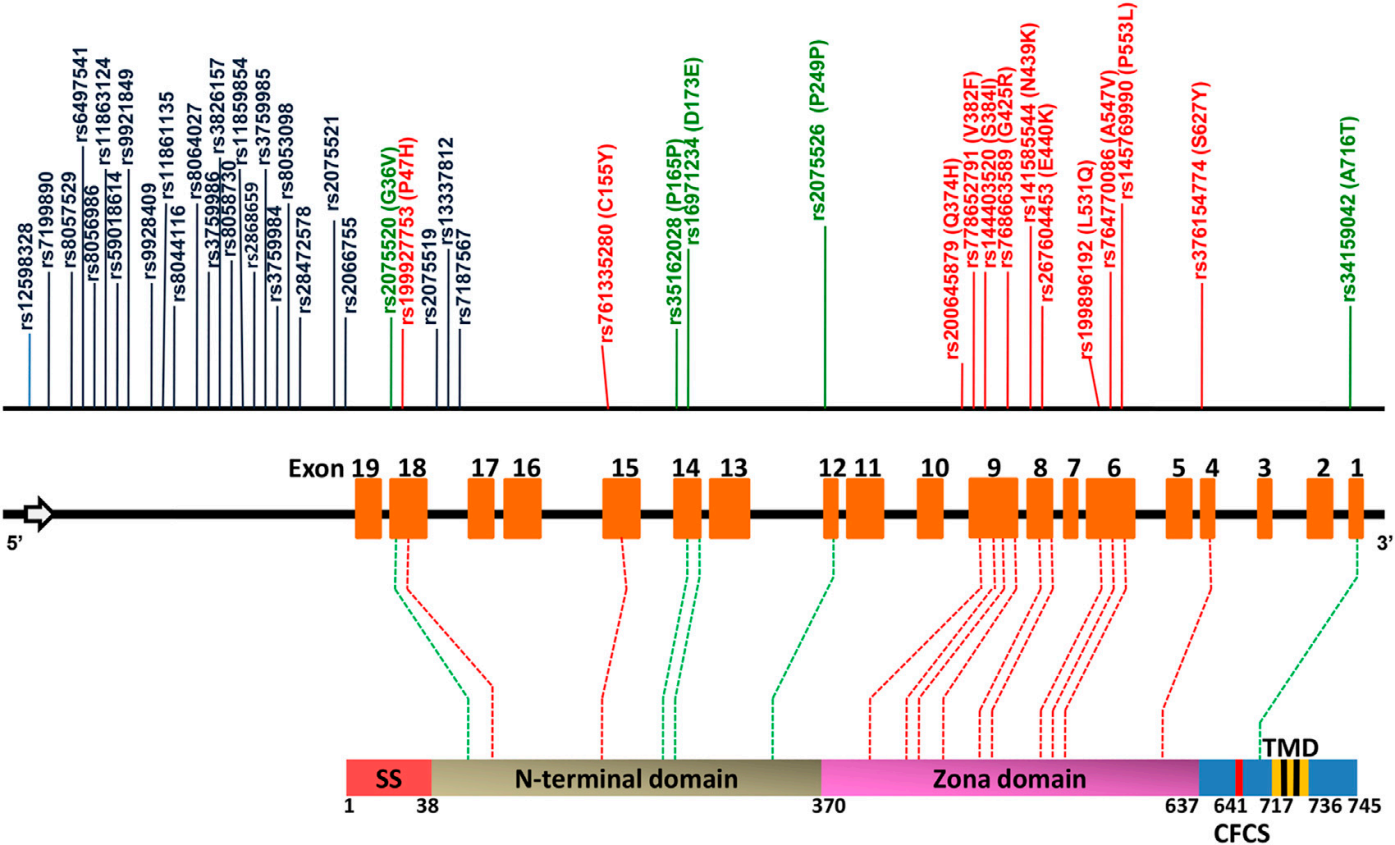
A map of *hZP2* gene and hZP2 protein, highlighting the positions of the various deleterious structural, functional and regulatory SNPs predicted by *in silico* analysis. Regulatory SNPs in the non-coding region are marked in black while those in the coding region are marked in green. Deleterious nsSNPs are marked in red. The sperm binding domain (aa 51–149) is present within the N-terminal domain. CFCS = consensus furin cleavage site; SS = signal sequence; TMD = transmembrane domain.

Female infertility is a major issue that is usually associated with hormonal or physiological issues. However, loss of function in any of the zona proteins due to SNPs may be another contributing factor (Pökkylä et al., 2011; Huang et al., 2014; Liu et al., 2017). The availability of a vast amount of genomic data and various bioinformatics algorithms makes it possible to shortlist the SNP’s which may affect the protein structure and function and hence female fertility. Using *in silico* analysis, we identified 12 deleterious nsSNPs, out of a total of 1,069 SNPs, which cause structural and/or functional changes. Among these 12, two are located within the N-terminal domain of hZP2 and the remaining 10 are located within the highly conserved zona domain of the protein (Figure 5). Previous studies have demonstrated that the N-terminal region of ZP2 protein (39–154 aa) is crucial for the initial zona-sperm interaction and the zona domain (372–631 aa) participates in the structural integrity of the zona matrix by regulating the polymerization of ZP proteins (Jovine et al., 2005; Baibakov et al., 2012; Avella et al., 2014).

The two nsSNPs present in the N-terminal region associated with zona-sperm interaction are rs199927753 and rs761335280. In rs199927753 (P47H) the small, non-reactive amino acid Pro is altered to His, a polar amino acid that can transfer protons on and off with ease, and in rs761335280 (C155Y), a Cys is substituted with a hydrophobic Tyr whose reactive hydroxyl group now makes it more likely for it to be involved in interactions with non-carbon atoms which was not earlier possible with Cys. These changes are predicted to affect the zona-sperm interaction **(** Table 7 **)**. It is important to note that the C155Y position is highly conserved suggesting its importance in this process.

**TABLE 7:** Summary of the properties of shortlisted deleterious nsSNPs and their effect.

S.No	rsIDs	Substitution	Conserved or not-conserved	Domain/Region of hZP2	Alterations	Probable reason
SNPs in the N-term region of hZP2						
1	rs199927753	P47H	Not Conserved	N terminal binding domain	Changes in interaction between sperm and egg	P: Small non-reactive; H: Polar, can transfer protons on and off with ease
2	rs761335280	C155Y	Conserved	N terminal binding domain	Changes in interaction between sperm and egg	Y has a reactive hydroxyl group; more involved in interactions with non-carbon atoms
SNPs in the Zona domain of hZP2						
3	rs200645879	Q374H	Not Conserved	Zona domain	Structural changes	H can easily move protons on and off its side chain as compared to Q
4	rs778652791	V382F	Not Conserved	Zona domain	Structural changes	Disfavored substitution as F is aromatic
5	rs144403520	S384I	Not Conserved	Zona domain	Structural changes	Disfavored substitution. I: hydrophobic; remains buried within the protein’s core
6	rs768663589	G425R	Conserved	Zona domain; metal binding	Structural changes	Disfavored substitution. G: sometimes plays a functional role in protein structures by providing a sidechain-less backbone to bind phosphates or other ligands. Gain in ADP-ribosylation at G425 and disulfide linkage at C424
7	rs141585544	N439K	Conserved	Zona domain	Structural changes	Predicted to decrease protein stability although both are polar amino acids; altered stability; loss of catalytic site at N439
8	rs267604453	E440K	Conserved	Zona domain	Structural changes	E: negatively charged. K: Positively charged. Most deleterious SNP; Probably affects interactions leading to structural changes
9	rs199896192	L531Q	Not Conserved	Zona domain	Structural changes	L: Non-polar. Q: Polar; prefers to be on the surface exposed to aqueous environment; gain of ADP-ribosylation at R533; altered stability
10	rs764770086	A547V	Conserved	Zona domain	Structural changes; metal binding	V: Longer c-beta branch leading to bulkiness in protein
11	rs145769990	P553L	Not Conserved	Zona domain	Structural changes	P: Highly exposed. L: Prefers to be buried inside protein’s core. Predicted deleterious by all 5 algorithmic tools
12	rs376154774	S627Y	Conserved	Zona domain	Structural integrity	Y: partially hydrophobic; prefers to be buried within the hydrophobic core; aromatic side chain may be involved in stacking interactions with other aromatic side chains; Predicted to form 1 new contact with Asp626. Can affect structural integrity

The remaining 10 nsSNPs are present in the zona domain of the protein and are probably affecting the structural integrity of the ZP matrix by altering the zona domain’s polymerization. This may be one of the background factors for causing various types of zona anomalies. The predicted structural changes due to amino acid substitutions in rs200645879 (Q374H), rs778652791 (V382F), rs144403520 (S384I), rs267604453 (E440K), rs768663589 (G425R), rs141585544 (N439K), rs199896192 (L531Q), rs764770086 (A547V), rs145769990 (P553L), and rs376154774 (S627Y) have been discussed in Table 7. Interestingly, the substitution in the SNP rs764770086 (A547V) is predicted to cause loss of disulfide linkage in the neighboring Cys (Cys^545^). The substitution in rs768663589 (G425R), on the other hand, predicts a gain of disulfide linkage at Cys^424^. ZP2 is rich in disulfide linkages and the gain or loss of these can cause structural changes affecting sperm-egg interaction.

Amongst these nsSNPs, the most deleterious SIFT and PolyPhen-2 score was observed with rs267604453 (E440K). Similarly, rs145769990 (P553L) seems to be an important SNP as it was predicted to be deleterious or disease linked by all the five algorithmic tools (SIFT, PolyPhen-2, PROVEAN, SNPs&GO, and PhD-SNP) that were used in the study (Table 1). Six of these (C155Y, G425R, N439K, E440K, A547V, and S627Y) are present in highly conserved positions.

ZP proteins are differentially glycosylated with Asn (N-) and Ser/Thr (O-) linked glycosylation. Several studies have implicated these glycans in either sperm-ZP interaction or in imparting structural characteristics to zona which makes the ZP available to the sperm receptors to bind to, or in imparting species specificity to this process (Yonezawa et al., 2007; Pang et al., 2011; Chiu et al., 2014; Clark, 2014). Based on these results, it can be hypothesized that the absence of glycans can result in changes that either affect the interaction between the egg and sperm, or its structure. In our predictions, we observed a changed glycosylation pattern for only O-glycans and except for S^631^, all other O-glycosylation sites which were lost were present downstream of aa 640. A propeptide corresponding to 641–745 aa is removed in mature hZP2. Hence, loss of these glycosylation sites will have no major effect either on sperm interaction or on the structure of the zona. Only S^631^ may be involved but that needs to be confirmed especially in the light of the fact that even though NetOGlyc predicted eight O-glycosylation sites for mouse ZP2 (S^9^, S^40^, T^626^, S^630^, S^633^, S^660^, S^666^, S^668^), mass spectrophotometric analysis on native mouse zona found only a single O-glycosylated site (T^455^) which was otherwise absent in the prediction (Boja et al., 2003).

In addition to these 12 deleterious nsSNPs, we also identified 31 regulatory SNPs that may affect the expression of the h*ZP2* gene at the transcription or translation level. A total of 26 regulatory SNPs (out of 31) are present in the non-coding region of the gene and are predicted to affect the transcription factor binding sites (TFBS) (Table 6 **)**. These SNPs were found to have a high global MAF value which signifies their occurrence in the population at a high frequency. Five SNPs (rs16971234, rs2075520, rs2075526, rs34159042, and rs35162028) from the coding region were predicted to affect splicing by acting either as exonic splicing enhancers (ESE) or exonic splicing silencers (ESS). These splicing regulatory elements (ESE or ESS) function by enrolling *trans*-acting splicing elements which can enhance or suppress the splice-site recognition and/or spliceosome assembly by various mechanisms resulting in a different mRNA transcript (Matlin et al., 2005). Most of these SNPs are located in the 5′ near gene region or in introns close to the 5′end of the gene.

It was interesting to find that among the five coding regulatory SNPs, one with rsID rs16971234 encodes for the substitution of Asp at position 173 with Glu. This nsSNP has a global MAF value of 0.2831 as per 1,000 genome project validation which points towards the high occurrence of this polymorphism in the population. The SNP is present in the N-terminal region which has been recognized as the cleavage site for the metalloprotease Ovastacin (Burkart et al., 2012). Altered splicing due to this SNP can result in a change in the cleavage site because of which Ovastacin cannot act. Transgenic mice studies in which the cleavage site was altered resulted in eggs where the ZP2 could not be cleaved post-fertilization and sperm continued to bind (Gahlay et al., 2010). These females were also found to have very low fertility rates. Also, this is a highly conserved position among mammals (Burkart et al., 2012). It will be interesting to study if this polymorphism in females also results in infertility. Two of the other predicted regulatory SNPs rs2075521 and rs2075526 have also been identified in a study conducted on three women with recurrent oocyte lysis during their IVF attempts (Ferré et al., 2014). Another regulatory SNP rs2075520 has been found in another study on patients with zona anomalies (Pökkylä et al., 2011). Thus, these studies support our results regarding the association of predicted deleterious SNPs with the reproductive fitness of females.

It has been hypothesized that multiple low-affinity binding sites may be involved in oocyte-sperm interaction, as this may be the natural way of preventing complete fertilization failure by numerous *de novo* generated nucleotide changes (Castle, 2002). Thus, it is possible that at the population level, several SNPs individually or in combination with others may affect fertility. However, further, experimental investigations are necessary to confirm the deleterious status of these SNPs at the population level. The identification and characterization of these will help in explaining the etiology behind various types of zona anomalies and unexplained infertility in human females and could potentiate their use in the diagnosis of female infertility.

## Data Availability

The datasets presented in this study can be found in online repositories. The names of the repository/repositories and accession number(s) can be found in the article/Supplementary Material.

## Supplementary Material

Supplementary Figure 1 A graphical representation of global MAF values of 31 SNPs predicted to effect the regulation of hZP2 by FuncPred. These 31 SNPs include 5 coding (green) and 26 non-coding SNPs (blue).
