# Supplementary figures and images for: Identification and in silico Characterization of Deleterious Single Nucleotide Variations in Human ZP2 Gene
Source: Front Cell Dev Biol. 2021 Nov 17;9:763166. doi: 10.3389/fcell.2021.763166 (PMC8635754; doi:10.3389/fcell.2021.763166)

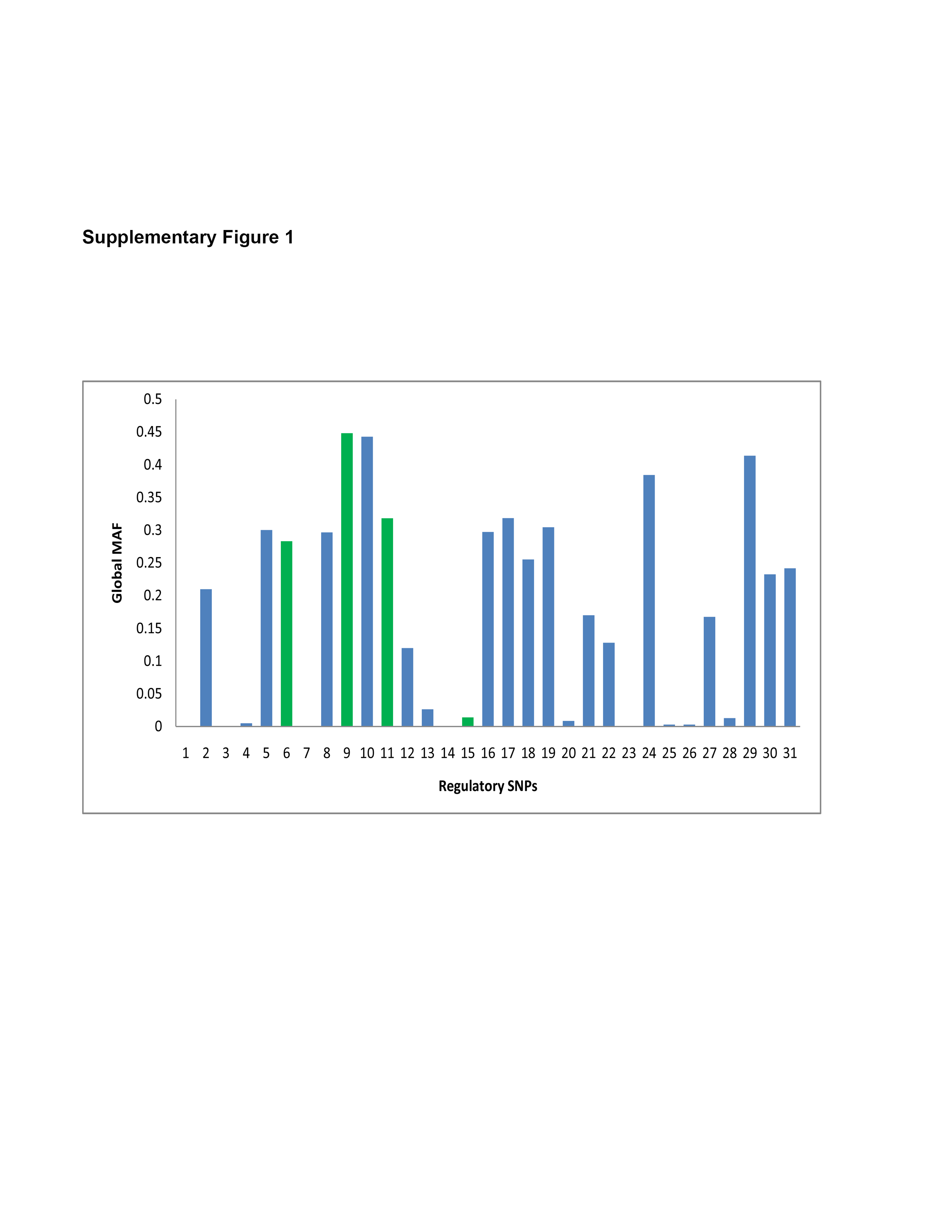

Supplement: Supplementary file 1 [file Image1.TIF]
