# Supplementary material for: Identification and in silico Characterization of Deleterious Single Nucleotide Variations in Human ZP2 Gene
Source: Front Cell Dev Biol. 2021 Nov 17;9:763166. doi: 10.3389/fcell.2021.763166 (PMC8635754; doi:10.3389/fcell.2021.763166)
Supplement: Supplementary file 2 [file DataSheet1.docx]

Supplementary Material

Supplementary Table 1: List of 22 deleterious nsSNPs in *hZP2* as per SIFT prediction. Those with a SIFT score of ≤0.05 were predicted to be deleterious.

| **S.No.** | **rsID** | **Residue change** | **SIFT Score** | **Prediction** |
| --- | --- | --- | --- | --- |
| 1. | rs202135737 | Q5E | 0.034 | Deleterious |
| 2. | rs188803389 | S25W | 0.038 | Deleterious |
| 3. | rs199927753 | P47H | 0.006 | Deleterious |
| 4. | rs367878569 | H74R | 0.031 | Deleterious |
| 5. | rs369985819 | D84N | 0.007 | Deleterious |
| 6. | rs141680915 | F159S | 0.046 | Deleterious |
| 7. | rs375240682 | I186T | 0.013 | Deleterious |
| 8. | rs369091148 | G282 | 0.003 | Deleterious |
| 9. | rs200645879 | Q374H | 0.045 | Deleterious |
| 10. | rs144403520 | S384I | 0.005 | Deleterious |
| 11. | rs373653232 | I419L | 0.028 | Deleterious |
| 12. | rs267604453* | E440K | 0 | Deleterious |
| 13. | rs186370257 | D447E | 0.007 | Deleterious |
| 14. | rs374388107* | T462I | 0 | Deleterious |
| 15. | rs147988348 | F493I | 0.014 | Deleterious |
| 16. | rs202104941 | S504F | 0.034 | Deleterious |
| 17. | rs202174621 | M526V | 0.014 | Deleterious |
| 18. | rs199896192 | L531Q | 0.001 | Deleterious |
| 19. | rs145769990 | P553L | 0.046 | Deleterious |
| 20. | rs140925075 | G581S | 0.015 | Deleterious |
| 21. | rs376154774 | S627Y | 0.03 | Deleterious |
| 22 | rs199787774 | D676G | 0.014 | Deleterious |

*SNPs with lowest SIFT score depicting more confidence in their prediction of being deleterious

**Supplementary Table 2:** List of 64 nsSNPs shown to have a damaging effect on hZP2 as per PolyPhen2 predictions. “Probably damaging” SNPs have a PolyPhen score between 0.950 and 1 and “Possibly damaging” have a PolyPhen score between 0.850 and 0.950.

| **S.No.** | **rsIDs** | **Residue change** | **PolyPhen2 Score** | **Prediction** |
| --- | --- | --- | --- | --- |
| 1. | rs573749216 | A2V | 0.952 | Possibly damaging |
| 2. | rs771310939 | W10C | 0.804 | Possibly damaging |
| 3. | rs373646750 | A18V | 0.608 | Possibly damaging |
| 4. | rs535254918 | F30S | 0.999 | Probably damaging |
| 5. | rs763331580 | T34S | 0.911 | Possibly damaging |
| 6. | rs199927753 | P47H | 0.998 | Probably damaging |
| 7. | rs559249999 | P50S | 0.894 | Possibly damaging |
| 8. | rs369985819 | D84N | 0.639 | Possibly damaging |
| 9. | rs376162002 | N105D | 0.565 | Possibly damaging |
| 10. | rs757369785 | A140P | 0.984 | Probably damaging |
| 11. | rs761335280* | C155Y | 1 | Probably damaging |
| 12. | rs190651334 | D158E | 0.704 | Possibly damaging |
| 13. | rs143028903 | R166W | 0.998 | Probably damaging |
| 14. | rs16971234 | D173E | 0.999 | Probably damaging |
| 15. | rs375240682 | I186T | 0.565 | Possibly damaging |
| 16. | rs752909556 | A262T | 0.615 | Possibly damaging |
| 17. | rs753003426 | P265T | 0.967 | Probably damaging |
| 18. | rs369091148* | G282E | 1 | Probably damaging |
| 19. | rs574632482 | N293Y | 0.972 | Probably damaging |
| 20. | rs767456177 | I304V | 0.791 | Possibly damaging |
| 21. | rs576630648 | L306P | 0.919 | Possibly damaging |
| 22. | rs373386450 | L314F | 0.989 | Probably damaging |
| 23. | rs761273317 | C329Y | 0.999 | Probably damaging |
| 24. | rs531980914 | R346W | 0.587 | Possibly damaging |
| 25. | rs375882969 | V350I | 0.488 | Possibly damaging |
| 26. | rs151236566 | V364I | 0.918 | Possibly damaging |
| 27. | rs142277591 | I366T | 0.956 | Possibly damaging |
| 28. | rs200645879* | Q374H | 1 | Probably damaging |
| 29. | rs774816416* | G376A | 1 | Probably damaging |
| 30. | rs763245616 | M378V | 0.999 | Probably damaging |
| 31. | rs778652791* | V382F | 1 | Probably damaging |
| 32. | rs144403520 | S384I | 0.957 | Probably damaging |
| 33. | rs755733475 | T395S | 0.997 | Probably damaging |
| 34. | rs763479712 | R416Q | 0.524 | Possibly damaging |
| 35. | rs373653232 | I419L | 0.941 | Possibly damaging |
| 36. | rs765444754* | P420T | 1 | Probably damaging |
| 37. | rs768663589* | G425R | 1 | Probably damaging |
| 38. | rs534452053 | T426M | 0.991 | Probably damaging |
| 39. | rs141585544* | N439K | 1 | Probably damaging |
| 40. | rs267604453* | E440K | 1 | Probably damaging |
| 41. | rs539699509 | H442Q | 0.537 | Possibly damaging |
| 42. | rs186370257 | D447E | 0.929 | Possibly damaging |
| 43. | rs374388107 | T462I | 0.999 | Probably damaging |
| 44. | rs760475024 | P484T | 0.866 | Possibly damaging |
| 45. | rs776951625 | E513K | 0.942 | Possibly damaging |
| 46. | rs772193478* | R521C | 1 | Probably damaging |
| 47. | rs779162964* | R521H | 1 | Probably damaging |
| 48. | rs199896192 | L531Q | 0.907 | Possibly damaging |
| 49. | rs764770086* | A547V | 1 | Probably damaging |
| 50. | rs145769990 | P553L | 0.985 | Probably damaging |
| 51. | rs367625701 | N560K | 0.596 | Possibly damaging |
| 52. | rs149764747 | V563M | 0.593 | Possibly damaging |
| 53. | rs373582627 | Y568D | 0.945 | Possibly damaging |
| 54. | rs140925075 | G581S | 0.937 | Possibly damaging |
| 55. | rs763474569 | V584M | 0.974 | Possibly damaging |
| 56. | rs149277141 | H586N | 0.945 | Possibly damaging |
| 57. | rs376154774 | S627Y | 0.977 | Probably damaging |
| 58. | rs746263996 | P657Q | 0.454 | Possibly damaging |
| 59. | rs779302403 | L663M | 0.978 | Probably damaging |
| 60. | rs139759688 | S667Y | 0.734 | Possibly damaging |
| 61. | rs369785011 | G681E | 0.608 | Possibly damaging |
| 62. | rs561403538 | G692E | 0.622 | Possibly damaging |
| 63. | rs747158847 | G710R | 0.972 | Probably damaging |
| 64. | rs143939599 | D711G | 0.935 | Possibly damaging |

*SNPs with the highest PolyPhen score depicting more confidence in their prediction of being damaging SNPs.

**Supplementary Table 3:** List of 51 deleterious nsSNPs as per PROVEAN predictions. Those with a PROVEAN score less than the cut-off (-2.5) were predicted to be deleterious.

| **S.No.** | **rsID** | **Residue change** | **PROVEAN score**  **(cut-off = -2.5)** | **Prediction** |
| --- | --- | --- | --- | --- |
| 1. | rs535254918 | F30S | -3.229 | Deleterious |
| 2. | rs199927753 | P47H | -3.168 | Deleterious |
| 3. | rs559249999 | P50S | -4.240 | Deleterious |
| 4. | rs76073950 | W73L | -7.028 | Deleterious |
| 5. | rs755332082 | I91N | -2.982 | Deleterious |
| 6. | rs754924175 | H114R | -3.256 | Deleterious |
| 7. | rs761335280 | C155Y | -8.190 | Deleterious |
| 8. | rs16971234 | D173E | -2.635 | Deleterious |
| 9. | rs749254725 | G191D | -3.214 | Deleterious |
| 10. | rs758938301 | F207L | -3.325 | Deleterious |
| 11. | rs775595040 | Y230H | -3.117 | Deleterious |
| 12. | rs200100492 | Y238H | -3.025 | Deleterious |
| 13. | rs753003426 | P265T | -3.691 | Deleterious |
| 14. | rs369091148 | G282E | -5.977 | Deleterious |
| 15. | rs574632482 | N293Y | -3.535 | Deleterious |
| 16. | rs576630648 | L306P | -2.836 | Deleterious |
| 17. | rs200654965 | N310S | -2.571 | Deleterious |
| 18. | rs373386450 | L314F | -2.698 | Deleterious |
| 19. | rs761273317 | C329Y | -6.454 | Deleterious |
| 20. | rs200645879 | Q374H | -3.468 | Deleterious |
| 21. | rs774816416 | G376A | -5.569 | Deleterious |
| 22. | rs763245616 | M378V | -3.124 | Deleterious |
| 23. | rs778652791 | V382F | -4.574 | Deleterious |
| 24. | rs144403520 | S384I | -3.800 | Deleterious |
| 25. | rs765444754 | P420T | -6.797 | Deleterious |
| 26. | rs768663589 | G425R | -7.425 | Deleterious |
| 27. | rs534452053 | T426M | -5.401 | Deleterious |
| 28. | rs773028658 | F430L | -2.543 | Deleterious |
| 29. | rs141585544 | N439K | -5.491 | Deleterious |
| 30. | rs267604453 | E440K | -3.644 | Deleterious |
| 31. | rs539699509 | H442Q | -3.571 | Deleterious |
| 32. | rs374388107 | T462I | -4.579 | Deleterious |
| 33. | rs753879706 | P483S | -4.380 | Deleterious |
| 34. | rs760475024 | P484T | -6.018 | Deleterious |
| 35. | rs202104941 | S504F | -4.375 | Deleterious |
| 36. | rs80015211 | G510V | -2.547 | Deleterious |
| 37. | rs147824024 | F519V | -2.828 | Deleterious |
| 38. | rs772193478 | R521C | -6.299 | Deleterious |
| 39. | rs779162964 | R521H | -3.454 | Deleterious |
| 40. | rs199896192 | L531Q | -4.879 | Deleterious |
| 41. | rs764770086 | A547V | -3.661 | Deleterious |
| 42. | rs145769990 | P553L | -8.892 | Deleterious |
| 43. | rs367625701 | N560K | -2.791 | Deleterious |
| 44. | rs373582627 | Y568D | -7.479 | Deleterious |
| 45. | rs768677584 | Y573H | -3.159 | Deleterious |
| 46. | rs140925075 | G581S | -2.715 | Deleterious |
| 47. | rs149277141 | H586N | -3.368 | Deleterious |
| 48. | rs376154774 | S627Y | -3.344 | Deleterious |
| 49. | rs371254101 | T633S | -2.501 | Deleterious |
| 50. | rs758922115 | T653R | -2.572 | Deleterious |
| 51. | rs746263996 | P657Q | -3.170 | Deleterious |

**Supplementary Table 4:** List of 26 disease associated nsSNPs as per SNPs&GO predictions. Those with a probability value of >0.05 are predicted to be disease associated.

| **S.No.** | **rsID** | **Residue change** | **Probability** | **Prediction** |
| --- | --- | --- | --- | --- |
| 1. | rs188803389 | S25W | 0.655 | Disease associated |
| 2. | rs535254918 | F30S | 0.843 | Disease associated |
| 3. | rs559249999 | P50S | 0.679 | Disease associated |
| 4. | rs76073950 | W73L | 0.506 | Disease associated |
| 5. | rs755332082 | I91N | 0.567 | Disease associated |
| 6. | rs754924175 | H114R | 0.534 | Disease associated |
| 7. | rs761335280 | C155Y | 0.754 | Disease associated |
| 8. | rs775595040 | Y230H | 0.697 | Disease associated |
| 9. | rs200100492 | Y238H | 0.726 | Disease associated |
| 10. | rs369091148 | G282E | 0.695 | Disease associated |
| 11. | rs761273317 | C329Y | 0.619 | Disease associated |
| 12. | rs774816416 | G376A | 0.576 | Disease associated |
| 13. | rs763245616 | M378V | 0.587 | Disease associated |
| 14. | rs778652791 | V382F | 0.599 | Disease associated |
| 15. | rs144403520 | S384I | 0.598 | Disease associated |
| 16. | rs765444754 | P420T | 0.621 | Disease associated |
| 17. | rs768663589 | G425R | 0.842 | Disease associated |
| 18. | rs141585544 | N439K | 0.805 | Disease associated |
| 19. | rs267604453 | E440K | 0.688 | Disease associated |
| 20. | rs539699509 | H442Q | 0.688 | Disease associated |
| 21. | rs772193478 | R521C | 0.670 | Disease associated |
| 22. | rs779162964 | R521H | 0.553 | Disease associated |
| 23. | rs199896192 | L531Q | 0.667 | Disease associated |
| 24. | rs764770086 | A547V | 0.623 | Disease associated |
| 25. | rs145769990 | P553L | 0.719 | Disease associated |
| 26. | rs373582627 | Y568D | 0.726 | Disease associated |

**Supplementary Table 5**: List of 54 disease associated SNPs as per PhD-SNP predictions. A Probability Score of ≥0.50 is labeled as disease associated while a score of <0.50 is labeled as neutral.

| **S.No.** | **rsID** | **Residue change** | **Reliability index** | **Probability score** | **Prediction** |
| --- | --- | --- | --- | --- | --- |
| 1. | rs771310939 | W10C | 5 | 0.758 | Disease associated |
| 2. | rs373646750 | A18V | 3 | 0.627 | Disease associated |
| 3. | rs188803389 | S25W | 9 | 0.943 | Disease associated |
| 4. | rs535254918 | F30S | 8 | 0.906 | Disease associated |
| 5. | rs763331580 | T34S | 4 | 0.696 | Disease associated |
| 6. | rs199927753 | P47H | 6 | 0.780 | Disease associated |
| 7. | rs559249999 | P50S | 5 | 0.748 | Disease associated |
| 8. | rs76073950 | W73L | 3 | 0.649 | Disease associated |
| 9. | rs755332082 | I91N | 4 | 0.699 | Disease associated |
| 10. | rs376162002 | N105D | 3 | 0.661 | Disease associated |
| 11. | rs764288775 | G113R | 0 | 0.504 | Disease associated |
| 12. | rs754924175 | H114R | 5 | 0.768 | Disease associated |
| 13. | Rs764730762 | A126T | 3 | 0.669 | Disease associated |
| 14. | rs757369785 | A140P | 5 | 0.763 | Disease associated |
| 15. | rs761335280 | C155Y | 7 | 0.845 | Disease associated |
| 16. | rs16971234 | D173E | 4 | 0.677 | Disease associated |
| 17. | rs749254725 | G191D | 6 | 0.803 | Disease associated |
| 18. | rs758938301 | F207L | 1 | 0.552 | Disease associated |
| 19. | rs150342177 | H219R | 2 | 0.601 | Disease associated |
| 20. | rs141308223 | N223S | 2 | 0.576 | Disease associated |
| 21. | rs775595040 | Y230H | 7 | 0.865 | Disease associated |
| 22. | rs200100492 | Y238H | 7 | 0.850 | Disease associated |
| 23. | rs755695168 | M239V | 1 | 0.541 | Disease associated |
| 24. | rs541487118 | S256P | 2 | 0.612 | Disease associated |
| 25. | rs752909556 | A262T | 1 | 0.551 | Disease associated |
| 26. | rs753003426 | P265T | 0 | 0.511 | Disease associated |
| 27. | rs369091148 | G282E | 4 | 0.722 | Disease associated |
| 28. | rs574632482 | N293Y | 0 | 0.517 | Disease associated |
| 29. | rs576630648 | L306P | 4 | 0.723 | Disease associated |
| 30. | rs761273317 | C329Y | 7 | 0.863 | Disease associated |
| 31. | rs142277591 | I366T | 2 | 0.620 | Disease associated |
| 32. | rs774816416 | G376A | 3 | 0.666 | Disease associated |
| 33. | rs763245616 | M378V | 0 | 0.516 | Disease associated |
| 34. | rs778652791 | V382F | 5 | 0.748 | Disease associated |
| 35. | rs144403520 | S384I | 0 | 0.500 | Disease associated |
| 36. | rs765444754 | P420T | 2 | 0.588 | Disease associated |
| 37. | rs768663589 | G425R | 9 | 0.926 | Disease associated |
| 38. | rs534452053 | T426M | 7 | 0.830 | Disease associated |
| 39. | rs773028658 | F430L | 0 | 0.515 | Disease associated |
| 40. | rs769526020 | E431K | 0 | 0.517 | Disease associated |
| 41. | rs141585544 | N439K | 8 | 0.903 | Disease associated |
| 42. | rs267604453 | E440K | 1 | 0.54 | Disease associated |
| 43. | rs776951625 | E513K | 1 | 0.555 | Disease associated |
| 44. | rs772193478 | R521C | 7 | 0.852 | Disease associated |
| 45. | rs779162964 | R521H | 6 | 0.812 | Disease associated |
| 46. | rs370008514 | R529T | 0 | 0.502 | Disease associated |
| 47. | rs199896192 | L531Q | 5 | 0.728 | Disease associated |
| 48. | rs764770086 | A547V | 5 | 0.734 | Disease associated |
| 49. | rs145769990 | P553L | 4 | 0.705 | Disease associated |
| 50. | rs373582627 | Y568D | 7 | 0.834 | Disease associated |
| 51. | rs768677584 | Y573H | 5 | 0.757 | Disease associated |
| 52. | rs376154774 | S627Y | 1 | 0.571 | Disease associated |
| 53. | rs747158847 | G710R | 1 | 0.569 | Disease associated |
| 54. | rs763462776 | V727A | 2 | 0.581 | Disease associated |

**Supplementary Table 6:** 18 shortlisted deleterious nsSNPs and their SNAP2 predictions

| **S.No.** | **rsID** | **Residue change** | **Prediction effect** | **Score** | **Expected accuracy** |
| --- | --- | --- | --- | --- | --- |
| 1. | rs199927753 | P47H | Effect | 44 | 71% |
| 2. | rs559249999 | P50S | Effect | 44 | 71% |
| 3. | rs761335280 | C155Y | Effect | 92 | 95% |
| 4. | rs369091148 | G282E | Effect | 73 | 85% |
| 5. | rs200645879 | Q374H | Effect | 44 | 71% |
| 6. | rs774816416 | G376A | Effect | 34 | 66% |
| 7. | rs778652791 | V382F | Effect | 52 | 75% |
| 8. | rs144403520 | S384I | Effect | 16 | 59% |
| 9. | rs765444754 | P420T | Effect | 10 | 59% |
| 10. | rs768663589 | G425R | Effect | 79 | 85% |
| 11. | rs141585544 | N439K | Effect | 80 | 91% |
| 12. | rs267604453 | E440K | Effect | 50 | 75% |
| 13. | rs374388107 | T462I | Effect | 28 | 63% |
| 14. | rs199896192 | L531Q | Effect | 3 | 53% |
| 15. | rs764770086 | A547V | Effect | 56 | 75% |
| 16. | rs145769990 | P553L | Effect | 33 | 66% |
| 17. | rs140925075 | G581S | Effect | 59 | 75% |
| 18. | rs376154774 | S627Y | Effect | 67 | 80% |
